# Emotion recognition in evolving facial expressions: A matter of believing

**DOI:** 10.3389/fnbeh.2022.951974

**Published:** 2023-01-11

**Authors:** Michael Sonnberger, Carola Widmann, Denise Potthoff, Rüdiger J. Seitz, Raimund Kleiser

**Affiliations:** ^1^Institute of Neuroradiology, Kepler-University Linz, Linz, Austria; ^2^Department of Neurology, Center of Neurology and Neuropsychiatry, LVR-Klinikum Düsseldorf, Medical Faculty, Heinrich-Heine University Düsseldorf, Düsseldorf, Germany

**Keywords:** emotion, empathy, beliefs, face expression, fMRI

## Introduction

Since the enlightenment period, beliefs have been considered widely as incompatible with science. This has promoted a reservation toward the notion of beliefs in the contemporary Western societies and the natural sciences, in particular. More recently, however, people have become aware that belief formation and believing can be a topic of increasing interest for a scientific discourse. Probably, this may have resulted from the observation that religious beliefs appeared as motivation for initiating outbursts of violence. It is important to realize, however, that beliefs are not limited to religious and political beliefs that are based on the narratives, but also comprise the so-called primal beliefs that do not depend on language functions as they concern objects and events in the environment (Seitz, 2022).

Believing is composed of cerebral processes involving the perception of external information and spontaneous appraisal of that information in terms of subjective value or meaning (Seitz et al., 2018). An important type of external information is human face, because facial expressions are considered as a human capacity to convey the emotional state of the given person (Russell, 1994). While the previous research on facial expressions of emotion has focused on the study of six basic categories, e.g., happiness, surprise, anger, sadness, fear, and disgust, recently, more than 20 compound facial expressions of emotions were identified that both can be produced and recognized as well (Du et al., 2014). In fact, humans are highly skilled to recognize the emotions in the rapidly changing facial expressions of other people (Fiske et al., 2007). Recognition of faces and facial expressions can be impaired in psychopathic disorders and alexithymia (Kyranides et al., 2022) as well as by the face masks that cover the nose and mouth (Kleiser et al., 2022). Importantly, however, the observing subject believes that she/he has recognized the facial expression of the other person and trust this belief (Brashier and Marsh, 2020). Moreover, upon recognition of the emotion in the facial expression of the other person, the facial muscles of the observing subject change in a corresponding fashion. This phenomenon demonstrated by electromyographic recordings was called facial mimicry (Franz et al., 2021). Accordingly, believing has an immediate impact on the expressive behavior of the believing subject (Seitz et al., 2022). The more pronounced the facial expressions are, the more likely the observing subject has recognized the observed emotion correctly and the more certain can she/he be in that belief.

Here, we requested healthy subjects to recognize the emotions in facial expressions that were displayed to them in video clips. Because we were interested in determining when the subjects believed to have recognized the emotions, we used video clips in which the emotional face expressions evolved within 20 s out of a neutral face. This allowed us to analyze the process of emotion recognition as compared to viewing the neutral face expressions and empathizing with the emotion seen in the face in the video clip. Empathy is the ability to take the other person's perspective that is considered a key element in entertaining interpersonal relationships (Bird and Viding, 2014). In a functional magnetic resonance imaging study, we found that recognition of an emotion in another person's facial expression results in the activation of large-scale cortico-subcortical circuits related to visual perception, emotion regulation, and action generation.

## Emotion recognition

Overall, 16 healthy subjects (8 females, 8 males, 25 ± 6 years, normal or corrected-to-normal vision) passed a screening for alexithymia (TAS-20, Bagby et al., 1994) and capability of empathy (SPF, http://psydok.sulb.unisaarland.de/volltexte/2009/2363/pdf/SPF_Artikel.pdf). They gave informed written consent to participate in the study that was approved by the local ethics committee and conducted according to the Declaration of Helsinki. Male and female facial expressions of happiness, sadness, fear, and anger consisted of depersonalized frontal black and white images (Averaged Karolinska Institute AKDEF). Each emotion starting from a neutral facial expression evolving over time up to the strongest expression of the emotion (30 images of 750 ms each) was presented. The subjects were instructed to press a button as soon as they recognized the emotion or felt that they empathized with the emotional expressions.

**Figure 1:**
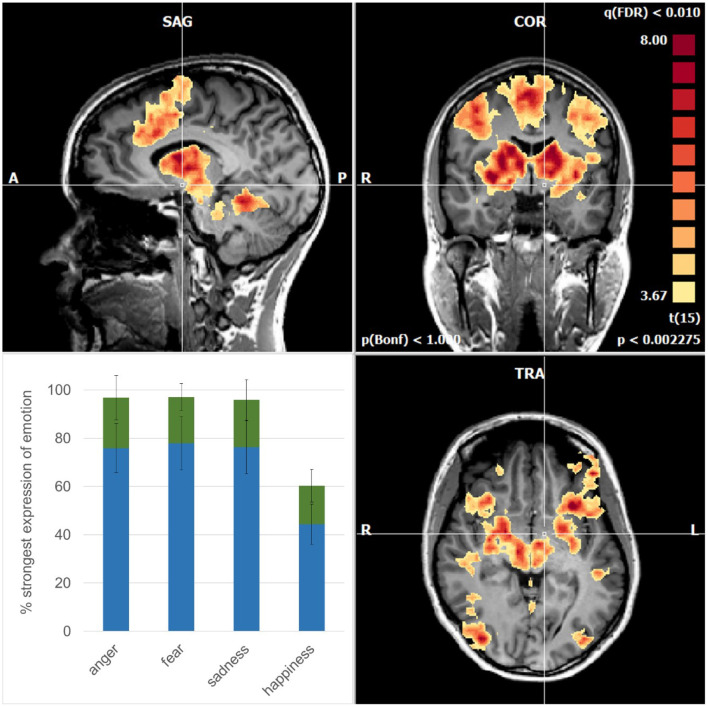
Mean activations are related to the recognition of the emotions in sagittal **(upper left)**, coronal **(upper right)**, and axial planes **(lower right)**. Note the symmetric pattern involves cortical areas and subcortical structures such as the basal ganglia, thalamus, amygdala, and brainstem nuclei. The cross of the stereotactic coordinates (x −4, y −4, z −6) signifies the left hypothalamus that is spared. **(lower left)** Degree of evolution of the emotional face expressions when the subjects recognized (blue) and empathized (green) with the emotions; error bars: standard deviations.

On average, the subjects recognized the emotion anger, fear, and sadness when each emotion had evolved in the video clips to ~80% (Figure 1). For comparison, happiness was recognized already when the emotion had evolved to some 40%, which is in accordance with the other studies (Adolphs, 2002). Interestingly, empathizing occurred while the facial expressions were still evolving with a similar delay across the four basic emotions (Figure 1).

## Brain activation

The focus of this functional magnetic resonance imaging (fMRI) study was to map the brain regions related to the processes of emotion recognition in stereotactic space (Talarairach and Tournoux, 1988). As validated in the brain of human primates, the changes in oxygenated blood as measured with fMRI are temporally and spatially related to the electrical field potential changes in neuronal assemblies following a definite sensory stimulus (Logothetis et al., 2001). fMRI was performed with a 3T MRI scanner (Siemens Magnetom Skyra) while the subjects were lying comfortably and viewed a mirror above them. Through this mirror, they observed the video clips. With a stimulation time of 22,500 ms and a control condition of 10,500 ms, this resulted in a total of 33,000 ms per block. A fixation cross was used as control stimulus shown between each trial to reset the BOLD signal (control condition). Each emotion was repeated six times, multiplied by four emotions in the two sexes resulting in a total of 48 repetitions and thus a total measurement time of 26.4 min. Whole-brain image analysis was done using the Brainvoyager QX software package version 21.4 (Brain Innovation, Maastricht, the Netherlands) as detailed elsewhere (Kleiser et al., 2017, 2022).

At that point when the subjects indicated by pressing a button to have recognized the emotions, there was a strong and widespread activation pattern involving cortical and subcortical brain structures (Figure 1). For comparison, before this time point, the activations were of a far weaker intensity (*p* < 0.05; FDR-corrected) occurring in brain areas related to the ventral pathway for the processing of shape, color, and faces, extending to V4, and to the fusiform gyrus (Courtney and Ungerleider, 1997). In addition, the dorsal pathway, related to the processing of motion such as visual area V5, the lateral parietal cortex, and the frontal eye fields were involved. In the phase, when the subjects indicated empathy with the emotions, there were activations (*p* < 0.05; FDR-corrected) as known to be activated in empathic processing such as the inferior frontal gyrus and the superior temporal gyrus (Shamay-Tsoory et al., 2009; Schurz et al., 2017). It is well possible that the subjects differed in how intensively and how long they were able to maintain empathy.

Importantly, however, the activations at the time point of recognition of the emotions exceeded an even higher level of significance (*p* < 0.01; FDR-corrected). In the cerebral cortex, they involved the dorsomedial frontal cortex including the supplementary motor area (SMA) and pre-SMA, the dorsolateral frontal cortex, the inferior frontal cortex, and the inferior temporal cortex in a virtually symmetric pattern in both cerebral hemispheres (Figure 1). In addition, there were strong activations in the basal ganglia, the entire thalamus, and amygdala and midbrain nuclei including the red nucleus. Finally, there was an involvement in the cerebellar vermis. These activations are compatible with the notion of an engagement of parallel cortico-subcortical cerebellar circuits (DeLong et al., 1984).

## Discussion

We have shown in the healthy subjects that believing to recognize the emotions in facial expressions engaged a widespread and distinctive network involving occipital, parietal, and frontal cortical areas. These areas are known to participate in the face recognition (Xu et al., 2021), oculomotor control (Pierrot-Deseilligny et al., 2004), and imitation of movement (Heiser et al., 2003). Moreover, strong activity was found also in subcortical structures such as the basal ganglia and the thalamus as a part of a highly developed cortico-subcortical relay circuitry, supporting these functions (DeLong et al., 1984). Furthermore, activity was found in the amygdala—a central neural component in emotion processing (Packard et al., 2021). Notably, this widespread pattern of cortical, subcortical, and cerebellar activations was similar to that recently observed in 944 participants during memory encoding of emotional pictures (Fastenrath et al., 2022). It is tempting to speculate that such an extended pattern of enhanced brain activity reflects the complexity of cerebral processing that may be suited to afford human conscious awareness (Greenfield and Collins, 2005).

With our experimental design, we were able to expand the duration of face presentation before the subjects recognized the emotion in the video clips. This allowed us to analyze the process of believing and to determine when the emotions were recognized correctly. It was amazing that this point occurred after the emotions were expressed to some 80%, and only happiness was recognized far earlier, corresponding to similar findings by others (Adolphs, 2002). The belief that the emotions were recognized correctly was substantiated subsequently when emotion was more pronounced. Thus, the subjects' trust in their correct recognition of the emotion was confirmed instantaneously in a rebound manner. This aspect probably also contributed to the strength of the activation pattern observed. In fact, these activations were far stronger than those related to viewing the faces that appeared neutral while empathizing with the emotional face expressions. Nevertheless, it was possibly the imaging that correlates with oscillatory binding of brain activity, when subjects become aware of information processing (Engel and Singer, 2001). That empathizing occurred only shortly later, suggesting that the awareness of the emotion was the bottle-neck process preceding empathizing. Moreover, subjective reports of the subjects support that the assumption empathizing with the emotions was strengthened by the slow progression into the emotion, as compared to an immediate exposure to an outspoken emotion upon viewing the static images of facial expressions.

People process sensory information with ease, which makes them susceptible to trusting these perceptions (Brashier and Marsh, 2020). Concerning affect recognition children, in contrast to adults, have been reported to observe both the eyes and the mouth (Guarnera et al., 2018). As happy faces typically have an open mouth that uncovers the teeth, this may serve as a clue for the observer to identify a happy emotion faster as compared to the other emotional states. The other basic emotions, such as sadness, anger, and fear, were recognized with similar ease (Kleiser et al., 2022). Importantly, however, humans believe that their perceptions are true reflections of the emotional states of the persons in their environment. This enables them to streamline the multitude of their sensory sensations according to those that are subjectively relevant for them and to select their subsequent behavioral actions accordingly (Seitz et al., 2022). Consequently, believing has not only a perceptive aspect about the subject's past experience, but also a prospective aspect concerning decision-making regarding the alternative actions with associated the predictions of what these actions will lead to and how the environment may react to these actions. The findings presented here provide empirical evidence for a putative neural basis for such processes of believing that afford intuitive, prelinguistic action generation (Seitz, 2022). Ultimately, they are apparently suited to support the concept that believing is a fundamental brain function (Angel and Seitz, 2016).

## Author contributions

RK and DP contributed to conception and design of the study. RK and CW carried out the measurements. RK, CW, and MS performed data post-processing and statistical analysis. RS, RK, and MS wrote sections of the manuscript. All authors contributed to manuscript revision, read, and approved the submitted version.
